# 1-Allyl-3-chloro-6-nitro-1*H*-indazole

**DOI:** 10.1107/S1600536809034138

**Published:** 2009-09-05

**Authors:** Nabil El Brahmi, Benchidmi Mohamed, El Mokhtar Essassi, Hafid Zouihri, Seik Weng Ng

**Affiliations:** a Laboratoire de Chimie Organique Hétérocyclique, Pôle de Compétences Pharmacochimie, Université Mohammed V-Agdal, BP 1014 Avenue Ibn Batout, Rabat, Morocco; b CNRST Division UATRS, Angle Allal Fassi/FAR, BP 8027 Hay Riad, 10000 Rabat, Morocco; c Department of Chemistry, University of Malaya, 50603 Kuala Lumpur, Malaysia

## Abstract

The indazole system in each of the two independent mol­ecules of the title compound, C_10_H_8_ClN_3_O_2_, is planar (r.m.s. deviations = 0.005 and 0.005 Å). The nitro group is coplanar with the fused-ring system [dihedral angles = 1.3 (3) and 4.8 (3) Å].

## Related literature

For a review of indazoles, see: Elguéro (1996 ▶); Elguéro *et al.* (1995 ▶).
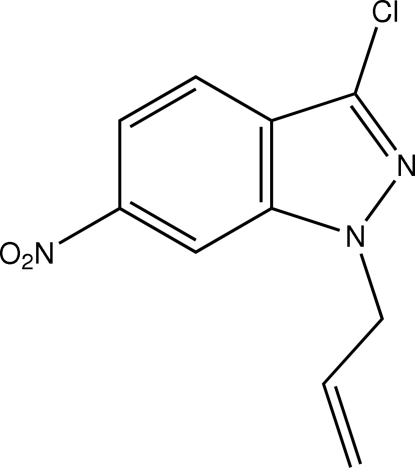


## Experimental

### 

#### Crystal data

C_10_H_8_ClN_3_O_2_
*M* *_r_* = 237.64
Monoclinic, 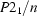

*a* = 7.6804 (1) Å
*b* = 9.9559 (2) Å
*c* = 28.4344 (4) Å
β = 95.144 (1)°
*V* = 2165.49 (6) Å^3^
*Z* = 8
Mo *K*α radiation
μ = 0.34 mm^−1^
*T* = 295 K
0.4 × 0.3 × 0.2 mm

#### Data collection

Bruker APEXII diffractometer
Absorption correction: multi-scan (*SADABS*; Sheldrick, 1996 ▶) *T*_min_ = 0.884, *T*_max_ = 0.934
19833 measured reflections
3777 independent reflections
2665 reflections with *I* > 2σ(*I*)
*R*_int_ = 0.032

#### Refinement

*R*[*F* ^2^ > 2σ(*F* ^2^)] = 0.056
*wR*(*F* ^2^) = 0.185
*S* = 1.07
3777 reflections
289 parameters
H-atom parameters constrained
Δρ_max_ = 0.78 e Å^−3^
Δρ_min_ = −0.29 e Å^−3^

### 

Data collection: *APEX2* (Bruker, 2005 ▶); cell refinement: *SAINT* (Bruker, 2005 ▶); data reduction: *SAINT*; program(s) used to solve structure: *SHELXS97* (Sheldrick, 2008 ▶); program(s) used to refine structure: *SHELXL97* (Sheldrick, 2008 ▶); molecular graphics: *X-SEED* (Barbour, 2001 ▶); software used to prepare material for publication: *publCIF* (Westrip, 2009 ▶).

## Supplementary Material

Crystal structure: contains datablocks global, I. DOI: 10.1107/S1600536809034138/tk2531sup1.cif

Structure factors: contains datablocks I. DOI: 10.1107/S1600536809034138/tk2531Isup2.hkl

Additional supplementary materials:  crystallographic information; 3D view; checkCIF report

## Figures and Tables

**Fig. 1.**
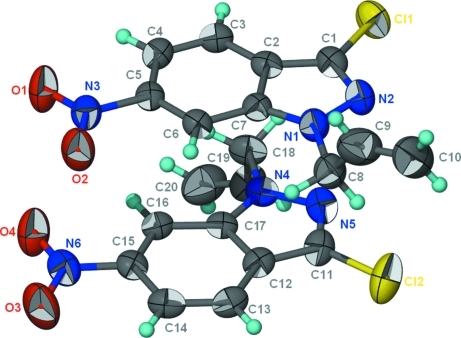
Thermal ellipsoid plot (Barbour, 2001) of the two independent molecules of C10H8ClN3O2 at the 50% probability level; hydrogen atoms are drawn as spheres of arbitrary radius.

## supplementary crystallographic information

### Experimental

3-Chloro-6-nitroindazole (5 mmol) and allyl bromide (10 mmol) were reacted in THF (40 ml) in the presence of potassium carbonate (10 mmol) and tetra-*n*-butylammonium bromide (0.5 mmol). The mixture was stirred for 24 h, filtered, and the THF removed under vacuum. The product was separated by chromatography on silica gel with a hexane:ethyl acetate (9:1) solvent system. The compound was obtained as yellow crystals in 50% yield; m.p. 351 K.

### Refinement

Carbon-bound H-atoms were placed in calculated positions (C—H 0.93 to 0.97 Å) and were included in the refinement in the riding model approximation with *U*(H) set to 1.2*U*(C).

Although data were measured to a high 2θ limit, those reflections beyond 50 ° were not used as their inclusion significantly raised the *R* index.

### Crystal data

**Table:**

C_10_H_8_ClN_3_O_2_	*F*(000) = 976
*M**_r_* = 237.64	*D*_x_ = 1.458 Mg m^−^^3^
Monoclinic, *P*2_1_/*n*	Mo *K*α radiation, λ = 0.71073 Å
Hall symbol: -P 2yn	Cell parameters from 6549 reflections
*a* = 7.6804 (1) Å	θ = 2.2–29.5°
*b* = 9.9559 (2) Å	µ = 0.34 mm^−^^1^
*c* = 28.4344 (4) Å	*T* = 295 K
β = 95.144 (1)°	Prism, yellow
*V* = 2165.49 (6) Å^3^	0.4 × 0.3 × 0.2 mm
*Z* = 8	

### Data collection

**Table:**

Bruker APEX2 diffractometer	3777 independent reflections
Radiation source: fine-focus sealed tube	2665 reflections with *I* > 2σ(*I*)
graphite	*R*_int_ = 0.032
φ and ω scans	θ_max_ = 25.0°, θ_min_ = 1.4°
Absorption correction: multi-scan (*SADABS*; Sheldrick, 1996)	*h* = −9→9
*T*_min_ = 0.884, *T*_max_ = 0.934	*k* = −11→11
19833 measured reflections	*l* = −33→33

### Refinement

**Table:**

Refinement on *F*^2^	Primary atom site location: structure-invariant direct methods
Least-squares matrix: full	Secondary atom site location: difference Fourier map
*R*[*F*^2^ > 2σ(*F*^2^)] = 0.056	Hydrogen site location: inferred from neighbouring sites
*wR*(*F*^2^) = 0.185	H-atom parameters constrained
*S* = 1.07	*w* = 1/[σ^2^(*F*_o_^2^) + (0.1031*P*)^2^ + 0.7179*P*] where *P* = (*F*_o_^2^ + 2*F*_c_^2^)/3
3777 reflections	(Δ/σ)_max_ = 0.001
289 parameters	Δρ_max_ = 0.78 e Å^−^^3^
0 restraints	Δρ_min_ = −0.29 e Å^−^^3^

### Fractional atomic coordinates and isotropic or equivalent isotropic displacement parameters (Å^2^)

**Table:**

	*x*	*y*	*z*	*U*_iso_*/*U*_eq_	
Cl1	0.30814 (12)	0.06578 (10)	0.03474 (3)	0.0718 (3)	
Cl2	0.94159 (18)	0.35838 (12)	0.02733 (4)	0.0958 (4)	
O1	0.6481 (4)	0.0762 (3)	0.28950 (9)	0.0866 (9)	
O2	0.7296 (4)	0.2788 (3)	0.27899 (9)	0.0867 (9)	
O3	1.2186 (4)	0.2329 (4)	0.28256 (10)	0.1089 (11)	
O4	1.0809 (4)	0.0460 (3)	0.27538 (10)	0.0928 (9)	
N1	0.5287 (3)	0.3527 (3)	0.10386 (9)	0.0563 (7)	
N2	0.4505 (4)	0.2960 (3)	0.06380 (9)	0.0606 (8)	
N3	0.6601 (4)	0.1750 (3)	0.26458 (9)	0.0582 (7)	
N4	0.8630 (3)	0.0501 (3)	0.09910 (10)	0.0529 (7)	
N5	0.8504 (4)	0.1220 (3)	0.05846 (10)	0.0622 (8)	
N6	1.1300 (4)	0.1509 (4)	0.25934 (11)	0.0659 (8)	
C1	0.4072 (4)	0.1742 (4)	0.07565 (11)	0.0523 (8)	
C2	0.4547 (3)	0.1450 (3)	0.12352 (11)	0.0451 (7)	
C3	0.4413 (4)	0.0352 (3)	0.15351 (11)	0.0509 (8)	
H3	0.3882	−0.0441	0.1424	0.061*	
C4	0.5079 (4)	0.0470 (3)	0.19951 (11)	0.0510 (8)	
H4	0.5003	−0.0244	0.2203	0.061*	
C5	0.5876 (4)	0.1675 (3)	0.21511 (10)	0.0458 (7)	
C6	0.6039 (4)	0.2781 (3)	0.18747 (10)	0.0447 (7)	
H6	0.6570	0.3568	0.1991	0.054*	
C7	0.5353 (4)	0.2648 (3)	0.14059 (11)	0.0447 (7)	
C8	0.6026 (5)	0.4877 (4)	0.10227 (14)	0.0678 (10)	
H8A	0.6737	0.5039	0.1317	0.081*	
H8B	0.6793	0.4909	0.0770	0.081*	
C9	0.4777 (6)	0.5939 (5)	0.0952 (2)	0.0994 (15)	
H9	0.4034	0.6041	0.1191	0.119*	
C10	0.4537 (7)	0.6752 (5)	0.0618 (2)	0.1091 (18)	
H10A	0.5232	0.6710	0.0366	0.131*	
H10B	0.3663	0.7398	0.0620	0.131*	
C11	0.9279 (4)	0.2371 (4)	0.06933 (11)	0.0588 (9)	
C12	0.9941 (4)	0.2448 (3)	0.11660 (11)	0.0488 (8)	
C13	1.0839 (4)	0.3397 (3)	0.14605 (13)	0.0563 (8)	
H13	1.1130	0.4232	0.1343	0.068*	
C14	1.1278 (4)	0.3072 (4)	0.19233 (13)	0.0563 (9)	
H14	1.1885	0.3681	0.2124	0.068*	
C15	1.0808 (4)	0.1816 (3)	0.20921 (11)	0.0491 (8)	
C16	0.9938 (3)	0.0852 (3)	0.18217 (11)	0.0453 (7)	
H16	0.9651	0.0023	0.1944	0.054*	
C17	0.9510 (3)	0.1194 (3)	0.13522 (11)	0.0435 (7)	
C18	0.8035 (4)	−0.0888 (3)	0.09845 (13)	0.0615 (9)	
H18A	0.6969	−0.0963	0.0776	0.074*	
H18B	0.7766	−0.1142	0.1299	0.074*	
C19	0.9352 (5)	−0.1820 (4)	0.08251 (14)	0.0701 (10)	
H19	0.9748	−0.1658	0.0531	0.084*	
C20	0.9988 (6)	−0.2813 (5)	0.1052 (2)	0.1019 (16)	
H20A	0.9629	−0.3012	0.1348	0.122*	
H20B	1.0817	−0.3350	0.0924	0.122*	

### Atomic displacement parameters (Å^2^)

**Table:**

	*U*^11^	*U*^22^	*U*^33^	*U*^12^	*U*^13^	*U*^23^
Cl1	0.0785 (6)	0.0739 (7)	0.0602 (6)	−0.0018 (5)	−0.0089 (4)	−0.0182 (4)
Cl2	0.1491 (11)	0.0756 (9)	0.0649 (6)	0.0153 (7)	0.0215 (6)	0.0187 (5)
O1	0.128 (2)	0.072 (2)	0.0577 (15)	0.0029 (16)	−0.0050 (15)	0.0184 (15)
O2	0.116 (2)	0.081 (2)	0.0593 (16)	−0.0218 (17)	−0.0108 (15)	−0.0064 (14)
O3	0.126 (2)	0.121 (3)	0.0733 (19)	−0.021 (2)	−0.0282 (18)	−0.0127 (19)
O4	0.133 (3)	0.074 (2)	0.0688 (18)	0.0066 (18)	−0.0030 (16)	0.0176 (16)
N1	0.0649 (16)	0.0493 (18)	0.0536 (16)	−0.0039 (13)	−0.0018 (12)	0.0018 (13)
N2	0.0677 (16)	0.063 (2)	0.0494 (16)	0.0022 (14)	−0.0031 (13)	−0.0010 (14)
N3	0.0651 (16)	0.060 (2)	0.0495 (16)	0.0074 (14)	0.0058 (13)	−0.0029 (15)
N4	0.0516 (14)	0.0464 (18)	0.0606 (17)	0.0000 (12)	0.0055 (12)	−0.0086 (13)
N5	0.0684 (17)	0.062 (2)	0.0560 (17)	0.0127 (15)	0.0025 (13)	−0.0061 (15)
N6	0.0645 (17)	0.071 (2)	0.0614 (19)	0.0134 (16)	−0.0007 (15)	−0.0084 (18)
C1	0.0503 (16)	0.053 (2)	0.0530 (19)	0.0056 (14)	0.0033 (14)	−0.0088 (16)
C2	0.0388 (14)	0.043 (2)	0.0543 (18)	0.0061 (12)	0.0063 (13)	−0.0087 (14)
C3	0.0506 (16)	0.041 (2)	0.062 (2)	0.0009 (13)	0.0101 (14)	−0.0064 (16)
C4	0.0567 (17)	0.042 (2)	0.0556 (19)	0.0068 (14)	0.0119 (15)	0.0031 (15)
C5	0.0443 (15)	0.047 (2)	0.0469 (17)	0.0094 (13)	0.0072 (13)	−0.0022 (14)
C6	0.0449 (15)	0.0386 (19)	0.0504 (17)	0.0050 (12)	0.0027 (13)	−0.0055 (14)
C7	0.0414 (14)	0.0427 (19)	0.0503 (17)	0.0079 (13)	0.0066 (12)	−0.0006 (15)
C8	0.078 (2)	0.054 (2)	0.069 (2)	−0.0089 (19)	−0.0050 (18)	0.0130 (18)
C9	0.085 (3)	0.078 (3)	0.134 (4)	−0.011 (2)	0.000 (3)	0.021 (3)
C10	0.108 (3)	0.080 (4)	0.132 (4)	−0.019 (3)	−0.023 (3)	0.046 (3)
C11	0.072 (2)	0.050 (2)	0.055 (2)	0.0154 (17)	0.0094 (17)	0.0019 (16)
C12	0.0496 (16)	0.040 (2)	0.0581 (18)	0.0094 (13)	0.0139 (14)	−0.0024 (15)
C13	0.0630 (19)	0.036 (2)	0.072 (2)	−0.0020 (14)	0.0176 (17)	−0.0019 (16)
C14	0.0510 (16)	0.047 (2)	0.072 (2)	−0.0019 (14)	0.0107 (15)	−0.0194 (17)
C15	0.0446 (15)	0.045 (2)	0.0577 (19)	0.0105 (13)	0.0057 (14)	−0.0034 (15)
C16	0.0460 (15)	0.0322 (18)	0.0589 (18)	0.0040 (12)	0.0105 (13)	0.0000 (14)
C17	0.0389 (14)	0.0354 (18)	0.0567 (18)	0.0060 (12)	0.0075 (13)	−0.0060 (14)
C18	0.0579 (18)	0.050 (2)	0.077 (2)	−0.0065 (15)	0.0049 (16)	−0.0166 (18)
C19	0.085 (2)	0.056 (3)	0.072 (2)	0.001 (2)	0.0204 (19)	−0.002 (2)
C20	0.096 (3)	0.082 (4)	0.131 (4)	0.013 (3)	0.030 (3)	0.027 (3)

### Geometric parameters (Å, °)

**Table:**

Cl1—C1	1.714 (3)	C6—H6	0.9300
Cl2—C11	1.708 (4)	C8—C9	1.430 (6)
O1—N3	1.221 (4)	C8—H8A	0.9700
O2—N3	1.218 (4)	C8—H8B	0.9700
O3—N6	1.219 (4)	C9—C10	1.250 (6)
O4—N6	1.213 (4)	C9—H9	0.9300
N1—C7	1.360 (4)	C10—H10A	0.9300
N1—N2	1.362 (4)	C10—H10B	0.9300
N1—C8	1.461 (4)	C11—C12	1.396 (4)
N2—C1	1.309 (5)	C12—C13	1.402 (5)
N3—C5	1.468 (4)	C12—C17	1.408 (4)
N4—N5	1.356 (4)	C13—C14	1.368 (5)
N4—C17	1.365 (4)	C13—H13	0.9300
N4—C18	1.456 (4)	C14—C15	1.398 (5)
N5—C11	1.316 (5)	C14—H14	0.9300
N6—C15	1.474 (4)	C15—C16	1.367 (4)
C1—C2	1.408 (4)	C16—C17	1.388 (4)
C2—C3	1.396 (4)	C16—H16	0.9300
C2—C7	1.410 (4)	C18—C19	1.474 (5)
C3—C4	1.366 (4)	C18—H18A	0.9700
C3—H3	0.9300	C18—H18B	0.9700
C4—C5	1.401 (4)	C19—C20	1.255 (6)
C4—H4	0.9300	C19—H19	0.9300
C5—C6	1.365 (4)	C20—H20A	0.9300
C6—C7	1.395 (4)	C20—H20B	0.9300
			
C7—N1—N2	111.0 (3)	H8A—C8—H8B	107.5
C7—N1—C8	128.9 (3)	C10—C9—C8	129.6 (6)
N2—N1—C8	119.9 (3)	C10—C9—H9	115.2
C1—N2—N1	105.8 (3)	C8—C9—H9	115.2
O2—N3—O1	122.8 (3)	C9—C10—H10A	120.0
O2—N3—C5	118.9 (3)	C9—C10—H10B	120.0
O1—N3—C5	118.3 (3)	H10A—C10—H10B	120.0
N5—N4—C17	111.6 (3)	N5—C11—C12	113.1 (3)
N5—N4—C18	119.5 (3)	N5—C11—Cl2	120.4 (3)
C17—N4—C18	128.6 (3)	C12—C11—Cl2	126.5 (3)
C11—N5—N4	105.3 (3)	C11—C12—C13	136.8 (3)
O4—N6—O3	123.4 (4)	C11—C12—C17	103.6 (3)
O4—N6—C15	118.8 (3)	C13—C12—C17	119.5 (3)
O3—N6—C15	117.8 (4)	C14—C13—C12	118.7 (3)
N2—C1—C2	113.0 (3)	C14—C13—H13	120.6
N2—C1—Cl1	121.1 (3)	C12—C13—H13	120.6
C2—C1—Cl1	125.9 (3)	C13—C14—C15	119.6 (3)
C3—C2—C1	136.5 (3)	C13—C14—H14	120.2
C3—C2—C7	120.4 (3)	C15—C14—H14	120.2
C1—C2—C7	103.1 (3)	C16—C15—C14	124.3 (3)
C4—C3—C2	118.4 (3)	C16—C15—N6	117.9 (3)
C4—C3—H3	120.8	C14—C15—N6	117.8 (3)
C2—C3—H3	120.8	C15—C16—C17	115.5 (3)
C3—C4—C5	119.5 (3)	C15—C16—H16	122.2
C3—C4—H4	120.3	C17—C16—H16	122.2
C5—C4—H4	120.3	N4—C17—C16	131.2 (3)
C6—C5—C4	124.7 (3)	N4—C17—C12	106.4 (3)
C6—C5—N3	117.7 (3)	C16—C17—C12	122.4 (3)
C4—C5—N3	117.7 (3)	N4—C18—C19	112.2 (3)
C5—C6—C7	115.3 (3)	N4—C18—H18A	109.2
C5—C6—H6	122.4	C19—C18—H18A	109.2
C7—C6—H6	122.4	N4—C18—H18B	109.2
N1—C7—C6	131.1 (3)	C19—C18—H18B	109.2
N1—C7—C2	107.2 (3)	H18A—C18—H18B	107.9
C6—C7—C2	121.8 (3)	C20—C19—C18	125.7 (4)
C9—C8—N1	115.3 (3)	C20—C19—H19	117.1
C9—C8—H8A	108.5	C18—C19—H19	117.1
N1—C8—H8A	108.5	C19—C20—H20A	120.0
C9—C8—H8B	108.5	C19—C20—H20B	120.0
N1—C8—H8B	108.5	H20A—C20—H20B	120.0
			
C7—N1—N2—C1	0.7 (3)	N2—N1—C8—C9	70.4 (5)
C8—N1—N2—C1	176.4 (3)	N1—C8—C9—C10	−116.9 (5)
C17—N4—N5—C11	−1.0 (3)	N4—N5—C11—C12	0.3 (4)
C18—N4—N5—C11	−174.4 (3)	N4—N5—C11—Cl2	179.8 (2)
N1—N2—C1—C2	−0.2 (4)	N5—C11—C12—C13	−179.6 (3)
N1—N2—C1—Cl1	−178.6 (2)	Cl2—C11—C12—C13	0.9 (6)
N2—C1—C2—C3	−179.7 (3)	N5—C11—C12—C17	0.4 (3)
Cl1—C1—C2—C3	−1.4 (5)	Cl2—C11—C12—C17	−179.0 (2)
N2—C1—C2—C7	−0.3 (3)	C11—C12—C13—C14	−179.7 (3)
Cl1—C1—C2—C7	178.1 (2)	C17—C12—C13—C14	0.2 (4)
C1—C2—C3—C4	179.3 (3)	C12—C13—C14—C15	−0.7 (4)
C7—C2—C3—C4	0.0 (4)	C13—C14—C15—C16	0.9 (5)
C2—C3—C4—C5	−0.3 (4)	C13—C14—C15—N6	−179.7 (3)
C3—C4—C5—C6	0.5 (4)	O4—N6—C15—C16	−5.2 (4)
C3—C4—C5—N3	−179.0 (3)	O3—N6—C15—C16	174.8 (3)
O2—N3—C5—C6	0.7 (4)	O4—N6—C15—C14	175.4 (3)
O1—N3—C5—C6	−179.1 (3)	O3—N6—C15—C14	−4.6 (4)
O2—N3—C5—C4	−179.8 (3)	C14—C15—C16—C17	−0.5 (4)
O1—N3—C5—C4	0.4 (4)	N6—C15—C16—C17	−179.9 (2)
C4—C5—C6—C7	−0.5 (4)	N5—N4—C17—C16	−180.0 (3)
N3—C5—C6—C7	179.0 (2)	C18—N4—C17—C16	−7.3 (5)
N2—N1—C7—C6	179.3 (3)	N5—N4—C17—C12	1.3 (3)
C8—N1—C7—C6	4.0 (5)	C18—N4—C17—C12	174.0 (3)
N2—N1—C7—C2	−0.8 (3)	C15—C16—C17—N4	−178.6 (3)
C8—N1—C7—C2	−176.1 (3)	C15—C16—C17—C12	0.0 (4)
C5—C6—C7—N1	−180.0 (3)	C11—C12—C17—N4	−1.0 (3)
C5—C6—C7—C2	0.2 (4)	C13—C12—C17—N4	179.0 (3)
C3—C2—C7—N1	−179.8 (3)	C11—C12—C17—C16	−179.9 (3)
C1—C2—C7—N1	0.7 (3)	C13—C12—C17—C16	0.2 (4)
C3—C2—C7—C6	0.1 (4)	N5—N4—C18—C19	81.6 (4)
C1—C2—C7—C6	−179.5 (3)	C17—N4—C18—C19	−90.5 (4)
C7—N1—C8—C9	−114.7 (4)	N4—C18—C19—C20	123.4 (5)
